# Inactivation of Staphylococcal Phenol Soluble Modulins by Serum Lipoprotein Particles

**DOI:** 10.1371/journal.ppat.1002606

**Published:** 2012-03-22

**Authors:** Bas G. J. Surewaard, Reindert Nijland, András N. Spaan, John A. W. Kruijtzer, Carla J. C. de Haas, Jos A. G. van Strijp

**Affiliations:** 1 Medical Microbiology, University Medical Center Utrecht, Utrecht, The Netherlands; 2 Department of Medicinal Chemistry and Chemical Biology, Institute for Pharmaceutical Sciences, Faculty of Science, Utrecht University, Utrecht, The Netherlands; Harvard Medical School, United States of America

## Abstract

*Staphylococcus aureus* virulence has been associated with the production of phenol soluble modulins (PSM). PSM are known to activate, attract and lyse neutrophils. However, the functional characterizations were generally performed in the absence of human serum. Here, we demonstrate that human serum can inhibit all the previously-described activities of PSM. We observed that serum can fully block both the cell lysis and FPR2 activation of neutrophils. We show a direct interaction between PSM and serum lipoproteins in human serum and whole blood. Subsequent analysis using purified high, low, and very low density lipoproteins (HDL, LDL, and VLDL) revealed that they indeed neutralize PSM. The lipoprotein HDL showed highest binding and antagonizing capacity for PSM. Furthermore, we show potential intracellular production of PSM by *S. aureus* upon phagocytosis by neutrophils, which opens a new area for exploration of the intracellular lytic capacity of PSM. Collectively, our data show that in a serum environment the function of PSM as important extracellular toxins should be reconsidered.

## Author Summary

Infections with methicillin-resistant *Staphylococcus aureus* (MRSA) are difficult to treat because of resistance against standard antibiotics. In contrast to the traditional healthcare-associated (HA-) MRSA strains, community-associated (CA-) MRSA strains cause severe infections in otherwise healthy individuals. CA-MRSA strains display enhanced virulence, spreading more rapidly and causing more severe illness than HA-MRSA strains. Enhanced virulence of CA-MRSA is thought to be associated with the production of several toxins, such as Phenol Soluble Modulins (PSM). PSM have been described to activate, attract and lyse neutrophils. Thus far, previous studies characterizing the functions of PSM were performed in the absence of body fluids. In the current study, we show that human serum strongly inhibits many functions attributed to PSM. We demonstrate that serum lipoprotein particles are responsible for the binding and inhibition of PSM, even when PSM are produced by growing *S. aureus* in whole blood. Finally, we show production of PSM by *S. aureus* within neutrophils, suggesting that PSM may play a role intracellularly in a serum-free environment. These findings significantly contribute to our understanding of the function of PSM and strongly suggest that PSM, instead of performing as extracellular toxins, most likely act as intracellular toxins.

## Introduction

*Staphylococcus aureus* frequently colonizes human anterior nares and can cause many infectious diseases, ranging from mild superficial skin and wound infections to life-threatening disseminated infections [1]. The number of infections by this bacterium is increasing, especially infections caused by methicillin-resistant *S. aureus* (MRSA) strains. However, infections are still limited to a small percentage of colonized individuals. This suggests that the human innate immune system together with physical and humoral barriers can very effectively control invasive infections, even those caused by the invasive community-associated (CA) MRSA. Therefore, we hypothesize that virulence factors produced by *S. aureus* are likely generally counteracted by the innate immune system, and that a balance between the two determines the outcome of an infection.

In order to survive within the host, *S. aureus* can make use of a variety of virulence factors [2], including a repertoire of toxins [3]. The toxins induce host cell lysis and include superantigens, leukocidins and phenol soluble modulins (PSM). In contrast to most other toxins, PSM are small core genome-encoded peptide toxins, except for PSM-*mec*, which is located on the methicillin resistance-encoding MGE staphylococcal cassette chromosome SCC*mec* [4]. The production of PSM is controlled by the quorum-sensing accessory gene regulator (*agr*) [5], and the gene expression levels correlate with strain virulence. Especially CA-MRSA strains are associated with high productions of PSM, which is thought to account for the enhanced virulence, easier spreading and severity of infection of CA-MRSA strains compared to hospital-acquired MRSA strains (HA-MRSA) [6], [7]. Thus far, all described PSM have a common amphipathic alpha helical region, which is thought to enable their cell lytic ability most likely by disrupting the cell membrane [7]. Despite having a similar structure, PSM are categorized in two groups, depending on their size. The smaller α-type PSM (PSMα1, PSMα2, PSMα3, PSMα4, and δ-toxin), with a length of 20–30 amino acids, are regarded as the most toxic PSM [7], whereas the larger β-type PSM (PSMβ1 and PSMβ2) of approximately 44 amino acids seem to have additional functions. For instance, β-type PSM of *S. epidermidis* are described to play a role in biofilm dispersal [8].

Next to lysing neutrophils, PSM are described to activate and attract leukocytes. Neutrophils are the first leukocytes recruited to the site of infection and are crucial in controlling staphylococcal infections. They are attracted by both host factors and conserved microbial molecules also known as pathogen-associated molecular patterns (PAMPs). Although many PAMPs are recognized by Toll-like receptors (TLRs) [9], PSM are potent staphylococci-specific PAMPs which act mainly on the human formylated peptide receptor 2 (FPR2) [10]. FPR2 is expressed on neutrophils, monocytes, macrophages, immature dendritic cells, and microglial cells, and its activation induces many neutrophil effector functions, including chemotaxis, exocytosis and superoxide generation [11]. While micromolar concentrations of PSM are needed for neutrophil lysis, nanomolar concentrations are enough for FPR2-mediated neutrophil stimulation. Although neutrophils sense PSM at nanomolar concentrations, *S. aureus* can subvert FPR2 signaling by producing the antagonists FPR2 inhibitory protein (FLIPr) [12] and its homologue FLIPr-like [13].

*S. aureus* typically resides in mucosal en epithelial surfaces and can invade beyond these physical barriers causing invasive infections. The switch from a colonizing phenotype to a virulent phenotype is regulated by *agr* [14]. This regulatory switch relies on the secretion of autoinducing peptide (AIP) sensed by the cell population and triggers the expression of virulence determinants such as proteases, haemolysins and toxins. To fight the infection, the human innate immune system has several effector mechanisms, both humoral and cellular, to clear the invading bacterium. Recently, Peterson et al [15] described a novel neutralizing mechanism; incorporating ApoB1 in VLDL and LDL lipoproteins in serum can sequester AIP and thereby disable staphylococcal quorum sensing. Mice lacking plasma ApoB1 are more susceptible to invasive staphylococcal infections, implicating that ApoB1 is an essential innate defense effector against *S. aureus* [15]. The current study shows that serum also provides a barrier against the pro-inflammatory and cytolytic activities of PSM, the highest *agr*-upregulated virulence factor of *S. aureus*. Our main finding is that PSM-induced activation and lysis of neutrophils are greatly inhibited by human serum. Lipoprotein particles were identified as the PSM-binding and -inhibiting components within serum, suggesting that they function as scavengers of PSM, thereby preventing host damage. We show that *S. aureus* potentially can produce PSM inside neutrophils after phagocytosis. This opens a new area of exploration of the intracellular toxic capacity of PSM.

## Results

### Activation and lysis of neutrophils by staphylococcal culture supernatants is inhibited by human serum

**Figure 1:**
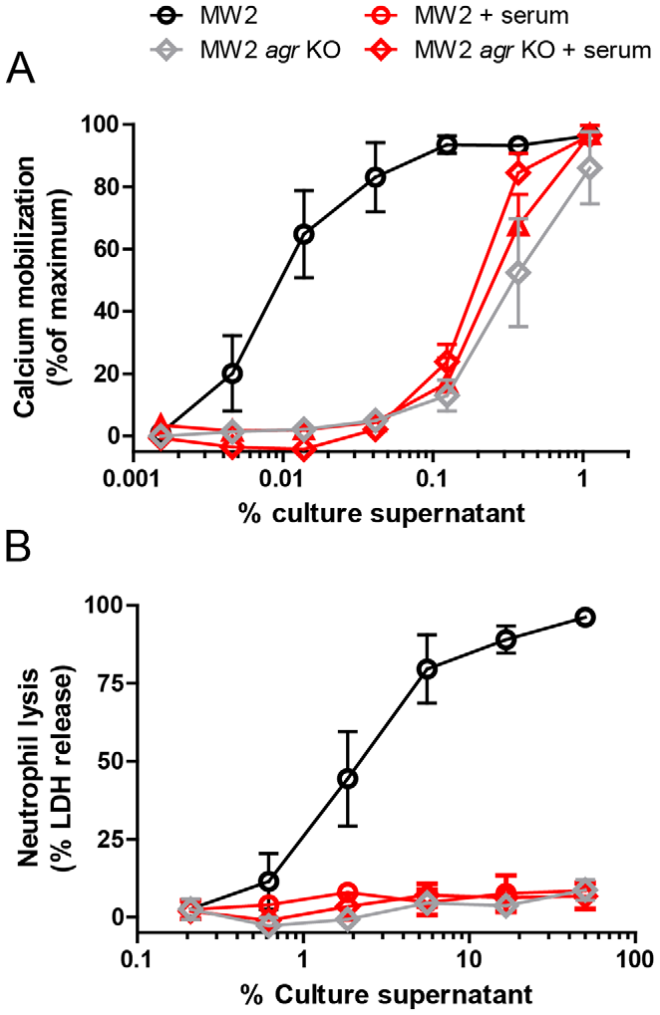
Human serum inhibits the activity of PSM in culture supernatants. (A) Dose-dependent calcium mobilization of HL-60/FPR2 cells by culture supernatants of *S. aureus* strains MW2 and MW2 *agr* KO, with or without preincubation with 1% heat-inactivated human serum. (B) Dose-dependent neutrophil lysis by *S. aureus* culture supernatants with or without preincubation with 5% heat-inactivated human serum. Neutrophil lysis was measured through LDH release. Data represent means ± SEM of three independent experiments.

Inflammatory PSM activities are generally studied in normal culture medium without the addition of serum [4], [7], [10], [16]. We hypothesized that constituents of human serum could inhibit *S. aureus* virulence by interfering with PSM activity at sites of infection. First we studied the influence of serum on the FPR2-stimulatory capacity of PSM produced by *S. aureus*. Therefore, we used an FPR2-transfected HL-60 cell line (HL-60/FPR2), and examined the activating capacity of culture supernatants of wild type (WT) MW2 strain and an isogenic *agr* knockout (MW2 *agr* KO) strain, which is described not to produce PSM [10] (Figure 1A). Without serum, the supernatant of WT MW2 activated HL-60/FPR2 cells very potently, in contrast to the supernatant of the MW2 *agr* KO strain. Addition of 1% normal human serum reduced the activity of the supernatant of WT MW2 to the level induced by the MW2 *agr* KO supernatant. The FPR2-activation by the MW2 *agr* KO supernatant was not affected by the addition of human serum, suggesting that human serum specifically inhibits the PSM-induced FPR2-activation. The inhibition of PSM-induced FPR2 activation by human serum was not species specific, as the addition of mouse, rabbit or bovine serum also inhibited the activation of HL-60/FPR2 cells by the WT MW2 supernatant (data not shown).

Next to the FPR2-activating capacity of *S. aureus* culture supernatants, the MW2 supernatant has also been described to very potently lyse isolated human neutrophils [7]. Therefore, we investigated the effect of serum on the culture supernatants of the same *S. aureus* strains in their capacity to lyse neutrophils. Indeed, the culture supernatant of WT MW2 very potently lysed human neutrophils, as measured by the release of LDH, in contrast to the supernatant of the MW2 *agr* KO (Figure 1B). Also in this assay, 5% human serum completely abrogated the lysis of human neutrophils induced by the culture supernatant of WT MW2, while leaving the effect of the MW2 *agr* KO supernatant uninfluenced. *Agr* not only regulates the production of PSM, but also controls the expression of other toxins, for instance the alpha toxin gene (hla). Based on the known functions of PSM, especially their FPR2 activating capacity, we hypothesize that PSM are the main effectors causing the differences in cell activation and lysis between the WT MW2 and the MW2 *agr* KO supernatants. Therefore, we think that PSM are specifically inhibited by human serum. As described by others [7], [10], we also observed that the percentage *S. aureus* MW2 supernatant needed for neutrophil lysis is 1000 times higher than that needed for FPR2 activation.

Not only *S. aureus* strain MW2, but also strains USA300 and Newman are known to produce high levels of PSM. In contrast, strains N315 and COL have been described to produce low levels of PSM [7], [10]. When we examined the activity of the supernats of these *S. aureus* strains, our results correlated with the described production of PSM. FPR2-induced cell activation and neutrophil lysis were only induced by the supernatants of the high PSM-producing *S. aureus* strains. Importantly, these activities were also potently inhibited by human serum (Figure S1A and S1B). Our findings thus suggest that human serum inhibits the FPR2-activation and neutrophil lysis induced by *S. aureus* produced PSM.

### Human serum inhibits the PSM-induced activation of neutrophils

**Figure 2:**
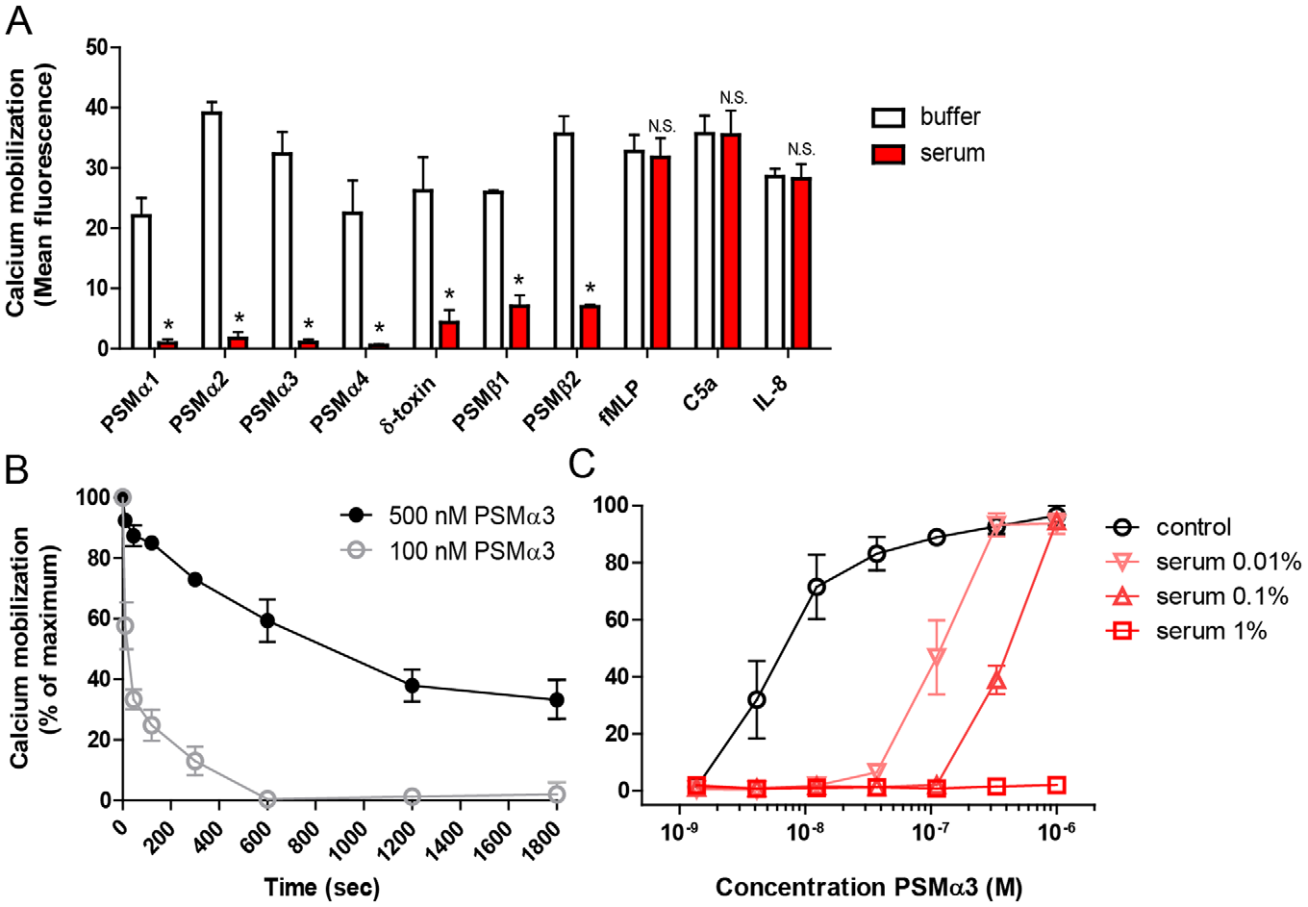
Human serum inhibits PSM-mediated neutrophil activation. (A) Calcium mobilization of human neutrophils. Neutrophils were stimulated with 10^−6^ M PSMα1, 10^−7^ M PSMα2, 10^−7^ M PSMα3, 10^−6^ M PSMα4, 3×10^−6^ M δ-toxin, 10^−5^ M PSMβ1, 10^−5^ M PSMβ2, 10^−9^ M fMLP, 10^−10^ M C5a and 10^−10^ M IL-8, all preincubated with or without 0.1% heat-inactivated human serum, before calcium mobilization was measured by flow cytometry. *, p<0.001; N.S., not significant. (B) Time-dependent inhibition of PSMα3-mediated calcium mobilization of HL60/FPR2 cells. PSMα3, 100 nM or 500 nM, was preincubated with 0.1% human serum and calcium mobilization was measured at different time-points by flow cytometry. (C) Dose-response curves for calcium mobilization in HL-60/FPR2 cells induced by PSMα3 or serum-treated PSMα3. Data represent means ± SEM of at least three independent experiments.

To demonstrate that human serum indeed targets PSM present in the supernatant of *S. aureus*, we also tested its activity on synthetic PSM. All *S. aureus* core genome-encoded PSM were tested, with the exception of PSM*mec*, for FPR2 stimulatory activity in the presence and absence of human serum. Pre-incubation of pure synthetic PSM with 0.1% human serum significantly inhibited the ability of all PSM to elicit calcium mobilization in neutrophils, whereas control stimuli, fMLP, IL-8 and C5a, were not inhibited (Figure 2A). The supernatant of *S. aureus* strain MRSA252 containing PSM*mec* was also inhibited by human serum. Although not examined for synthetic PSM*mec*, there is no reason to believe that human serum acts differently to PSM*mec* than to other PSM. To investigate the kinetics of serum-induced PSM-inactivation, the incubation time of PSM with serum was varied from 0 to 1800 sec, before testing in a calcium mobilization assay using HL-60/FPR2 cells. 100 nM PSMα3 was inactivated by 0.1% serum within seconds, whereas inactivation of a 5 times higher PSM concentration was clearly delayed (Figure 2B). Higher concentrations of PSM of up to 1 µM could be fully inhibited by 15 minutes pre-incubation with 1% serum (Figure 2C). These results indicate a time- and dose-dependent inhibition of PSM-induced FPR2-activation by human serum.

### Human serum inhibits the *S. aureus* PSM-induced neutrophil lysis

**Figure 3:**
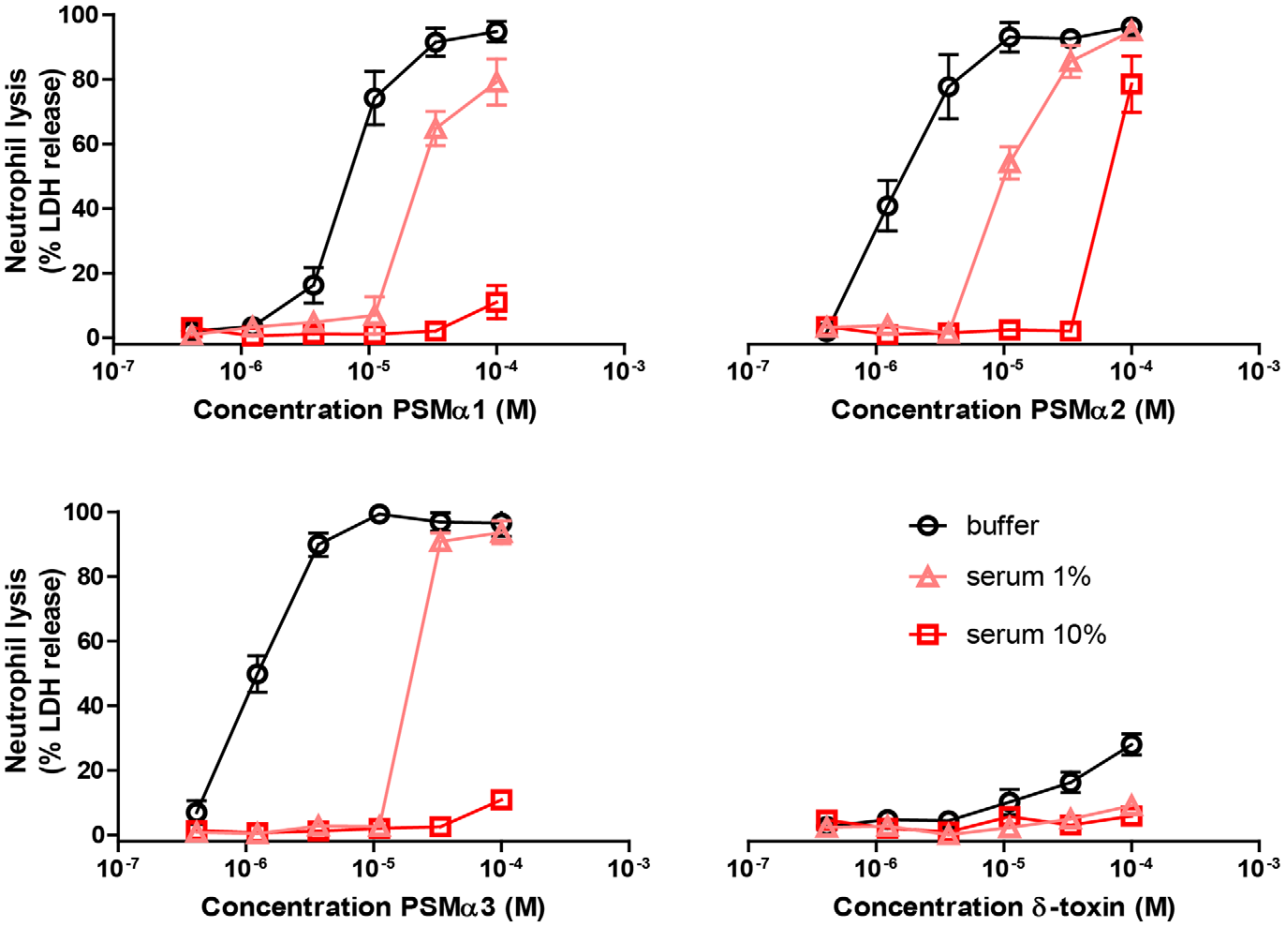
Inhibition of PSM-mediated neutrophil lysis. Dose-dependent neutrophil lysis by synthetic PSMα1, PSMα2, PSMα3, and δ-toxin (400 nM to 100 µM), preincubated with or without 1% or 10% human serum. Neutrophil lysis was measured through LDH release. Data represent means ± SEM of three independent experiments.

In order to investigate the ability of human serum to inhibit PSM-induced neutrophil lysis, we screened the synthetic PSM peptides preincubated with serum for neutrophil lysis. For this, we used a concentration range of 400 nM to 100 µM for each PSM. These concentrations are biologically relevant, as Wang et al [7] have described that the CA-MRSA MW2 and USA300 strains produce δ-toxin up to 30 µM in an overnight culture. Other α-type PSM are typically produced at somewhat lower concentrations, ranging from 5 µM to 15 µM. The cytolytic activity of PSMα1, PSMα2, PSMα3, and δ-toxin towards neutrophils was completely abrogated in the presence of serum (Figure 3). In addition, the lysis of peripheral blood mononuclear cells by synthetic PSMα3 was inhibited by human serum (Figure S2A). To investigate whether other staphylococcal toxins may also be inhibited by human serum, we tested the effect of serum on PVL-mediated lysis. The lysis of neutrophils induced by PVL toxin was not affected by human serum (Figure S2B), implicating that serum does not generally inhibit all toxin-mediated cell lysis. As shown previously [7], α-type PSM are very potent in cytolysis; however, in our hands neutrophil lysis by β-type PSM under physiologically-relevant concentrations was not observed. Collectively, we demonstrated that serum inhibits the FPR2-activating and neutrophil lysing capacity of all *S. aureus* PSM.

### PSM interact with serum lipoproteins

**Figure 4:**
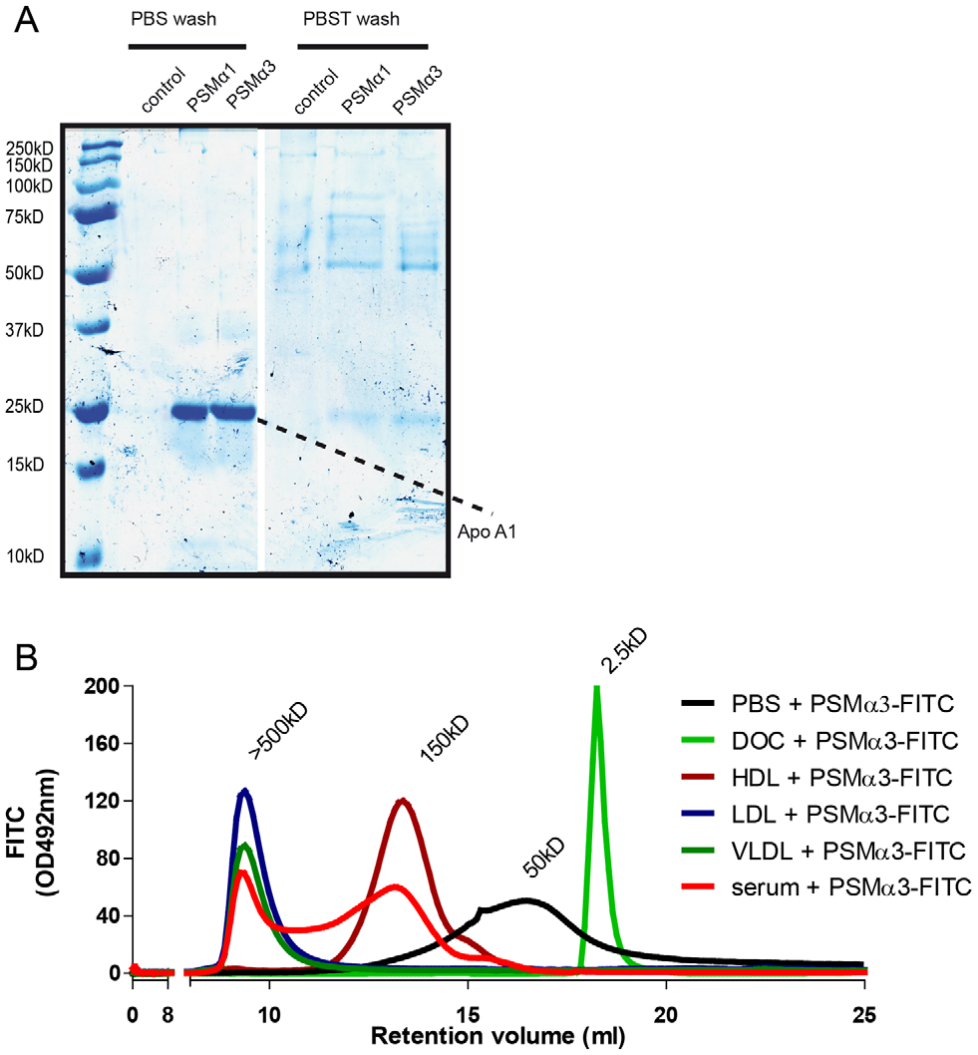
PSM associate with serum lipoproteins. (A) Serum pull down assay with PSMα1 and PSMα3 coupled to CNBr beads. Beads were extensively washed with PBS or PBS with tween (PBST). Serum proteins bound and eluted from the beads were visualized by SDS-page followed by instant blue staining. Protein bands specifically appearing in the PSMα1 and PSMα3 lane were identified by MALDI-TOF mass spectrometry as ApoA1. (B) Gel filtration association assay. Comparison of absorption (OD492 nm) profiles of 100 µg/ml PSMα3-FITC pre-incubated with PBS, 10% human serum, 1 mg/ml HDL, 1 mg/ml LDL or 1 mg/mL VLDL for 30 min, before separation on a gel filtration column. For monomerization of PSMα3-FITC, the gel filtration column was equilibrated with PBS containing 0.1% sodium deoxycholate (DOC). Representative figures of two independent experiments.

To capture the serum components able to inactivate PSM, we coated CNBr-Sepharose beads with synthetic *S. aureus* PSMα1 or PSMα3 and incubated the generated beads with 20% heat-inactivated serum. Following extensive washing, the specifically-bound proteins were eluted, analyzed by non-reducing SDS-PAGE and visualized by Instant Blue staining (Figure 4A). Both PSMα1 and PSMα3 bound to a protein of approximately 25 kDa, which was identified by mass spectrometry as ApoA1. Interestingly, when we performed the same experiment and additionally washed the PSM-coated beads after serum incubation with a detergent, ApoA1 was not longer detected. We were unable to detect a direct interaction of immobilized PSM with recombinant ApoA1 using ELISA or Surface Plasmon Resonance (data not shown). ApoA1 is however the major protein constituent of high density lipoprotein (HDL), which could possibly bind PSM. Serum lipoproteins are complex particles with a neutral core containing triglycerides and cholesterol and covered by an amphipathic monolayer of phospholipids and unesterified cholesterol. The Apo-protein components bind to the surface of the particles and are either restricted to particular lipoproteins or freely exchangeable across lipoprotein categories. Therefore, the inability of PSM to bind to ApoA1 in the presence of a detergent most likely indicates that PSM bind to the lipid content of the HDL particle rather than a specific interaction with ApoA1. To test this hypothesis, we performed serum size exclusion assays with FITC-labeled PSMα3 (PSMα3-FITC) to find the PSM-binding components in serum. The FITC-labeling did not affect the function of the PSMα3 molecule, as PSMα3-FITC was as potent as unlabeled PSMα3 in the FPR2-activation of neutrophils (Figure S3). At first, the retention volume of PSMα3-FITC was determined; PSMα3-FITC behaved as an aggregate of peptides in a physiological buffer, resulting in a broad peak of approximately 50 kDa (Figure 4B). In contrast, when a similar size exclusion run was performed in the presence of a detergent, a peak of monomeric PSMα3-FITC of approximately 2,5 kDa was observed. This is in line with the first-described discovery of PSM, where similar poly-peptide complexes were observed when PSM were extracted from *S. epidermidis* culture supernatant [17].

Multimeric aggregations have also been reported for *S. aureus* δ-toxin with molecular weights of 5–200 kDa, with the lower molecular weights observed at extreme pH or in organic solvents, and the larger molecular weights observed in water [18], [19]. Interestingly, when PSMα3-FITC was incubated with human serum prior to the size exclusion assay, the 50 kDa peak shifted towards two peaks, one of 150 kDa and one larger than 500 kDa, suggesting an association with HDL, known to run at 150 kDa, and low density (LDL) or very low density (VLDL) lipoproteins, known to run at higher than 500 kDa. To demonstrate that indeed PSMα3-FITC associated with lipoproteins in serum, we purified HDL and LDL from serum and repeated the size exclusion experiment using PSMα3-FITC preincubated with purified HDL or LDL. When PSMα3-FITC was preincubated with HDL, detection of the FITC signal shifted from a molecular weight of 50 kDa towards 150 kDa, whereas the signal of PSMα3-FITC preincubated with LDL, which, as VLDL, represents high molecular weight particles, shifted to the void volume of the gel filtration column. Importantly, the fluorescent 150 and >500 kDa peaks of the size exclusion chromatograms of PSMα3-FITC and serum correspond and are overlapping with the peaks of the size exclusion chromatograms of PSMα3-FITC with HDL and PSMα3-FITC with LDL or VLDL. As the interactions of PSMα3-FITC with HDL, LDL or VLDL do not shift the retention times of these lipoproteins on the gel filtration column, it appears that only monomeric PSMα3-FITC molecules interact with the lipoprotein particles (Figure S4). In conclusion, several serum lipoproteins can associate with PSM.

### Lipoproteins antagonize PSM

**Figure 5:**
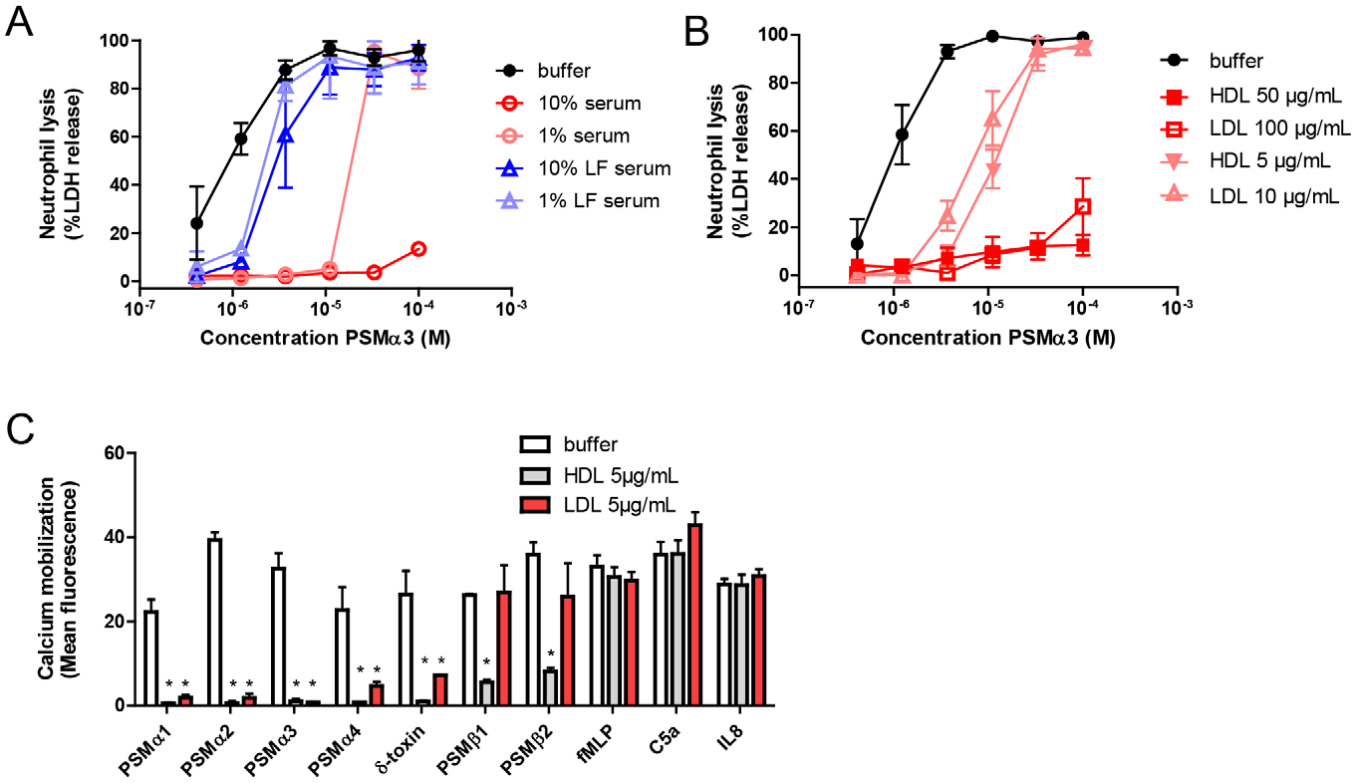
Serum lipoproteins inhibit PSM-mediated neutrophil lysis and activation. (A) Dose-dependent neutrophil lysis by synthetic PSMα3 preincubated with 1% and 10% human serum or human lipid-free (LF) serum or preincubated with (B) 5 and 50 µg/ml HDL or 10 and 100 µg/ml LDL (concentrations are based on protein content). PBS was used as buffer control. Neutrophil lysis was measured via LDH release. (C) Calcium mobilization of human neutrophils. Neutrophils were stimulated with 10^−6^ M PSMα1, 10^−7^ M PSMα2, 10^−7^ M PSMα3, 10^−6^ M PSMα4, 3×10^−6^ M δ-toxin, 10^−5^ M PSMβ1, 10^−5^ M PSMβ2, 10^−9^ M fMLP, 10^−10^ M C5a and 10^−10^ M IL-8, all preincubated with or without 5 µg/ml HDL or LDL, before calcium mobilization was measured by flow cytometry. *, p<0.001; N.S., not significant.

**Figure 6:**
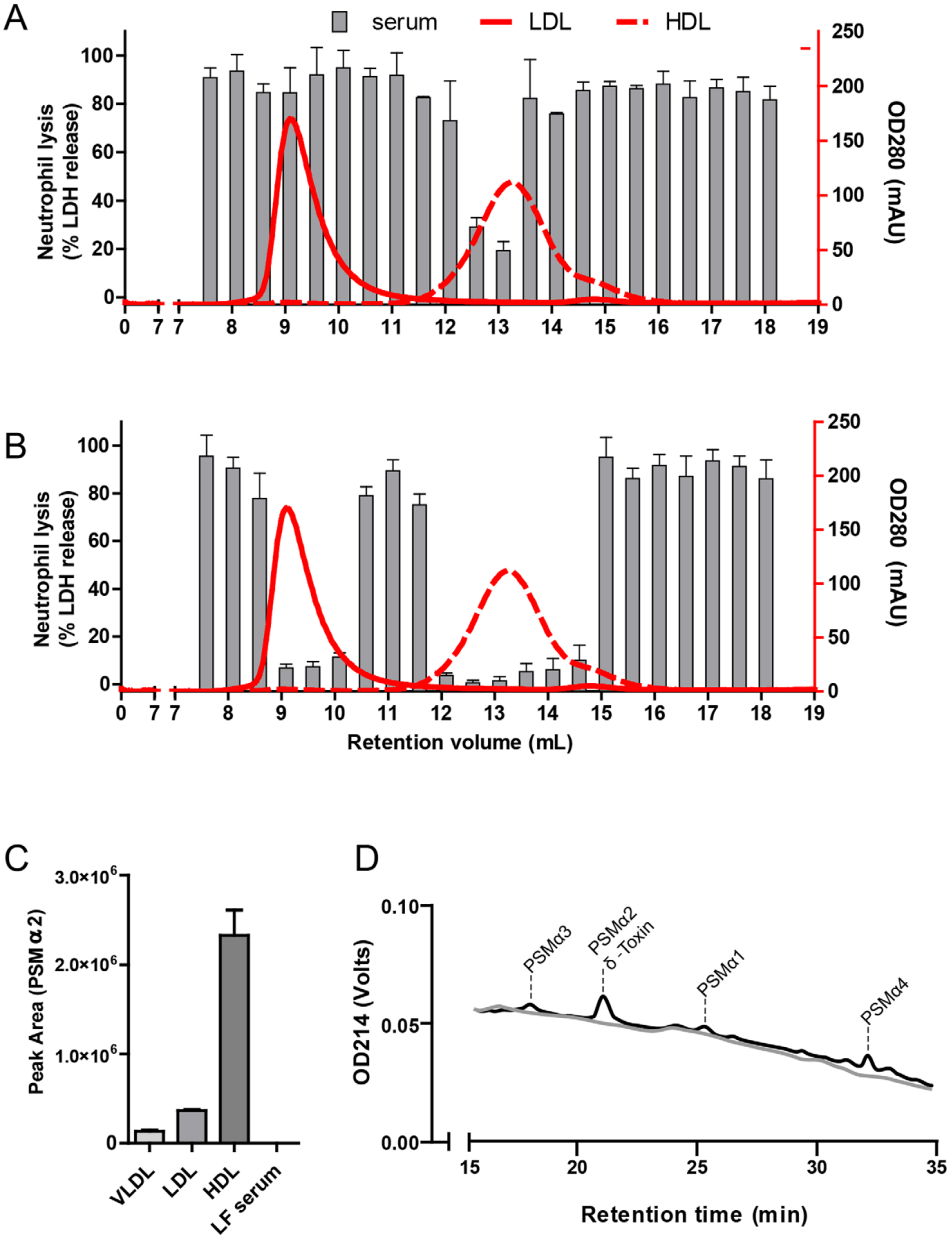
Identifying the most potent inhibitor of PSM in serum. Functional screening of serum fractions, isolated by gel filtration, for the inhibition of neutrophil lysis. Serum fractions were incubated with (A) 50 µM or (B) 10 µM of PSMα3 before addition to neutrophils and neutrophil lysis was measured via LDH release. Data represent means ± SEM of three independent experiments. (C) PSM concentration measured in isolated lipoprotein fractions after spiking human serum with 0.5 mg/ml PSMα2. PSM concentration was measured by reverse phase-HPLC and represents the mean of the area under the curve of PSMα2 of 3 independent experiments. (D) Measurement of the concentration PSM by HPLC in isolated HDL fraction from an overnight whole blood culture of the *S. aureus* MW2 strain (black line) or control (no bacteria; gray line). HDL fractions were subjected to HPLC and absorbance at 214 nm was obtained. Respective PSM were identified by LC/MS, δ-toxin and PSMα2 were contained in the same peak in this assay condition.

We further investigated whether serum lipoproteins, by binding PSM, are responsible for antagonizing the functions of PSM. Therefore, we depleted normal human serum for lipoproteins by density ultracentrifugation. Compared to untreated serum, lipoprotein-depleted serum lost the ability to inhibit the PSM induced lysis of neutrophils (Figure 5A), indicating that lipoproteins are indeed the serum components that inhibit PSM. Next, we tested the effect of purified HDL and LDL on PSM-function. Normal HDL or LDL protein levels in serum are between 1 and 1.3 mg/ml. Purified HDL and LDL at physiologically relevant concentrations, representing serum levels of 1 and 10%, inhibited the PSM-mediated lysis of neutrophils (Figure 5B). In addition, HDL and LDL inhibited the calcium mobilization induced by synthetic PSM (Figure 5C). Addition of recombinant ApoA1 or ApoB1 did not inhibit the PSM induced activation (Figure S5A) or lysis (Figure S5B) of neutrophils. To determine the serum lipoprotein with the highest neutralizing capacity, we separated human serum by size exclusion chromatography. Subsequently, the separate fractions were tested for inhibition of a high or a low lethal dose of PSMα3 (Figure 6). At the high lethal dose (50 µM) only the fractions containing HDL inhibited neutrophil lysis, whereas at a lower still lethal dose (10 µM) also LDL/VLDL could inhibit the lysis of neutrophils, indicating that HDL is the most potent inhibitor within human serum. Next, human serum was spiked with PSMα2, and HDL, LDL, VLDL and lipid-free serum were isolated by density ultracentrifugation and analyzed by HPLC (Figure 6C). Approximately 80% of the spiked PSMα2 was found in the HDL-containing fractions, whereas approximately 15% and 5% of PSM was recovered in LDL- and VLDL-containing fractions, respectively. We did not detect PSM in the lipid-free serum. Finally, we investigated whether PSM could also be recovered in serum constituents when *S. aureus* was cultured in blood. Therefore, we cultured *S. aureus* MW2 overnight in freshly drawn lepirudin-anticoagulated blood, isolated the HDL fraction from the clarified blood the following day and analyzed it by HPLC/LC MS. Figure 6D shows the presence of PSMα1, PSMα2, PSMα3, PSMα4 and δ-toxin in the purified HDL fraction, indicating that PSM produced by viable *S. aureus* could be neutralized by lipoproteins in a blood environment.

### PSMα is produced by *S. aureus* upon phagocytosis by neutrophils

**Figure 7:**
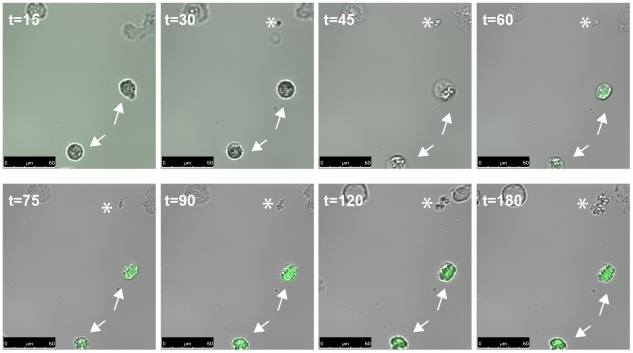
Rapid upregulation of PSMα expression after phagocytosis. *S. aureus* MW2 containing the PSMα promoter-GFP construct was incubated with neutrophils, and fluorescence as a measure for PSMα expression was monitored over time (0–3 hours). *Arrows* indicate neutrophils which have phagocytozed *S. aureus*. (*) Indicate growing reporter bacteria outside of the cells. Bars: 50 µm.

Thus far, our data strongly suggest that the functional properties of PSM produced by *S. aureus* in serum as well as in whole blood are inhibited by lipoproteins. These results conflict with literature describing PSM as key virulence determinants for CA-MRSA, with regard to their cell lytic properties [7]. Therefore, we hypothesized that PSM rather act as toxins in an environment devoid of lipoproteins, such as the neutrophil phagosome. To test whether PSM could act intracellular and are produced by *S. aureus* after uptake by human neutrophils, we generated a construct in which the promoter of the PSMα operon drives GFP expression. This PSMα promoter-GFP construct was transformed into *S. aureus* MW2, the bacterium was cultured over time, and the activation of the PSMα promoter was monitored with a fluorescent plate reader. As expected for an *agr*-controlled expression, the GFP expression was detected during the late logarithmic growth phase. Under these conditions, we did not observe expression of GFP when we introduced the construct in the MW2 *agr* KO strain (data not shown). Next, we examined GFP expression upon phagocytosis of MW2 bacteria containing the PSMα promoter-GFP construct by neutrophils. Therefore, the bacteria were first cultured to very early logarithmic phase to prevent initial activation of the PSMα promoter. Then, they were opsonized with human serum, presented to neutrophils adherent to a flow cell, and bacterial fluorescence upon phagocytosis by neutrophils was assayed over time with a fluorescent microscope. Fluorescence within neutrophils was observed 45 min to 2 hours after phagocytosis (Figure 7+Video S1), indicating activation of the PSMα promoter and thus potential production of PSMα inside neutrophils. We did not observe fluorescence for bacteria found outside neutrophils, except when they formed dense micro-colonies (data not shown). These data indicate potential PSMα expression inside neutrophils after phagocytosis of *S. aureus*.

## Discussion

Serum transports the humoral components of the innate immune system throughout the human body. Apart from the complement system and the coagulation system, the lipid transport system has been found to play a role in innate immunity as well. ApoB1, the major protein constituent of VLDL and LDL, can sequester AIP and thereby prevent quorum sensing of *S. aureus* [15]. We show in this study that serum lipoproteins dampen inflammatory over-stimulation and inhibit the cytolytic activities of the *agr*-controlled PSM. PSM are sequestered by serum lipoprotein particles and thereby lose their functional properties, which may allow their clearance from the system, analogous to LPS sequestration by HDL [20]. This has great implications for our understanding of PSM function during infection. All classes of plasma lipoproteins are normally present in interstitial tissue fluids at approximately 6% of the plasma concentration for VLDL to 20% for HDL [21]. This interstitial lipoprotein concentration even increases in case of inflammation. Although PSM can lyse neutrophils and *S. aureus* can produce PSM in very large quantities [6], [7], the majority of these PSM will most likely rapidly be neutralized by serum lipoproteins, making it unlikely that the high concentrations required to cause neutrophil lysis can be reached. Therefore, we propose that the role of PSM as important secreted extracellular toxins should be reconsidered.

We do not rule out a possible role for the membrane-lytic effects of PSM within neutrophils after phagocytosis of *S. aureus*. Intracellular lysis could account for the fact that experimental mouse studies with PSM knockout *S. aureus* strains show reduced virulence [4], [7]. It has recently been described that the concentration of AIP, responsible for gene transcription upon quorum sensing, can reach the critical concentration within cells, allowing the *agr* system to function within the neutrophil [22], [23]. In line with these studies, we show that the promoter for the PSMα operon is activated after phagocytosis of *S. aureus* by neutrophils. This strongly suggests that PSMα cytolytic peptides are produced within the neutrophil phagosome and may have a function there. Since the neutrophil phagosome is devoid of lipoprotein particles, PSM may act intraphagosomally and allow *S. aureus* to escape from the phagosome and thereby avoiding killing. Similarly, staphylococcal alpha toxin (Hla), has been demonstrated to allow endosomal escape after phagocytosis [24], [25]. However, further research is needed to test whether PSM have a similar mechanism of action. Next to the neutrophil phagosome, other niches within the human body lacking serum lipoproteins may allow for extracellular PSM functions.

PSM are thought to lyse cells by disrupting the cell membrane. *S. aureus* delta toxin is proposed to form a cation-selective membrane pore with a central hydrophilic channel by the multimerization of 6 monomers and an outer hydrophobic interaction with membrane lipid [18], [26]. The intrinsic structural properties of the amphipathic alpha helix of PSM likely mediate a similar multimerization in response to a lipid bilayer. This implies that PSM randomly insert into lipid membranes without specific targeting of host cells. In line with this, PSMγ and PSMδ from *S. epidermidis* have been shown to perforate synthetic POPC/POPG ((1-palmitoyl-2-oleoyl-*sn*-glycero-3-phosphocholine)/(1-palmitoyl-2-oleoyl-*sn*-glycero-3-[phospho-*rac*-(1-glycerol)]) lipid vesicles [27]. PSM thus seem to target lipid layers without the presence of membrane proteins, suggesting that there is no species specificity. This is in contrast to other staphylococcal cytolytic toxins, such as PVL, which show strong human specificity [28]. Staphylococcal two-component toxins are proposed to induce receptor-mediated lysis, which explains why they are not inhibited by human serum lipoproteins. PSM seem to have high affinity for lipids. Therefore, it is not surprising that their biological actions are inhibited by the major humoral lipid transportation system, the lipoprotein particles. The two most abundant serum lipoprotein particles are HDL and LDL. Although the normal serum concentration of LDL (ApoB1 1.6 mg/ml) is higher than the HDL (ApoA1 1.2 mg/ml), the combined surface of the HDL particles exceeds 3 times the surface of LDL particles. The surface size difference therefore most likely accounts for the higher inhibitory potential of HDL compared to LDL.

As we clearly show that PSM not only interact with HDL, but also with LDL and VLDL, we argue that the Apo proteins within the lipoprotein particles do not play a specific role in binding the PSM. Although we tried, we could indeed not detect a direct interaction between purified APO-proteins and synthetic PSM. In addition, PSM are unable to bind to lipoprotein particles in the presence of a detergent, and PSM can interact with synthetic lipid vesicles [27]. Therefore, we expect that PSM bind to the lipid contents of the lipoprotein particles without the need for a specific interaction with Apo proteins within the complexes.

PSM have the capacity of attracting neutrophils in the nanomolar range, exceeding their lytic capacity over a 1000 fold. There is increasing evidence that *S. aureus* can survive inside neutrophils [29] and that intracellular survival contributes to pathogenesis [30]. Neutrophil attraction to the site of infection may thus possibly be advantageous for the bacterium. On the one hand, it has been demonstrated that neutrophils are necessary to control the infection; to subvert neutrophil attraction through FPR2 *S. aureus* can also secrete two FPR2 antagonists, FLIPr or FLIPr-like [12], [13] and prevent excessive PSM-induced neutrophil migration. The higher PSM production of CA-MRSA strains, as compared to HA-MRSA strains, might however tilt the balance in favour of neutrophil migrating towards the infection side. Then, a higher PSM production inside the neutrophils upon *S. aureus* phagocytosis may rescue CA-MRSA from phagosomal killing, contributing to its enhanced virulence.

Serum lipoproteins have a well-known function in dampening immune responses. As for PSM, lipopolysaccharide (LPS) and lipoteichoic acid can be inactivated by human serum lipoproteins [31]–[33]. Also comparable to PSM, LPS forms large aggregates in aqueous conditions. Moreover, both PSM and LPS are taken up by HDL as monomeric molecules, resulting in their inactivation. The process of LPS- and PSM-inactivation by HDL displays different kinetics, since it takes hours for HDL to take up LPS [34], whereas we show uptake of PSM by HDL within seconds. For LPS, the serum components LPS-binding protein (LBP) and soluble CD14 (sCD14) catalyze the process of LPS transfer. Both LBP and sCD14 bind to the toxic Lipid A moiety of LPS and facilitate the transfer of monomeric LPS to HDL or to cellular expressed CD14, resulting in inactivation of LPS and activation of the LPS receptor TLR4, respectively. Thus far, there is no evidence that a similar serum transfer system exists for the transfer of PSM to lipoproteins or the recognition of PSM by FPR2. Our data show that isolated HDL, without the addition of other serum components, is sufficient for PSM transfer to HDL. Additionally, we show no enhancement of the FPR2 activating capacity of PSM in the presence of lipoprotein deficient serum, as compared to buffer only. These results strongly suggest that no serum component is needed for the PSM inactivation by lipoproteins or the PSM recognition by FPR2.

High production of PSM by CA-MRSA strains is proposed as the causative factor for the enhanced virulence of CA-MRSA strains, as compared to HA-MRSA strains [6], [7]. Our current study shows strong interaction and neutralization of PSM by serum lipoproteins, even when PSM are produced by growing *S. aureus* in whole blood. These results strongly suggest that the contribution of PSM to the enhanced virulence of CA-MRSA strains is not due to PSM acting as extracellular toxins. PSM can only function intracellular or in other lipoprotein-free niches in the body. Low non-toxic concentrations of PSM might attract neutrophils to the site of infection enabling uptake of *S. aureus*. Once inside the cell, production of PSM might help *S. aureus* in its escape from the phagosome, aiding in its survival and virulence. The antimicrobial activity of PSM against other bacteria might also create a niche for staphylococcal colonization outside the human body. Future studies are needed to shed more light on the exact functions of PSM and their contributions to CA-MRSA virulence.

## Materials and Methods

### Ethics statement

Informed written consent was obtained from all donors and was provided in accordance with the Declaration of Helsinki. Approval was obtained from the medical ethics committee of the University Medical Center Utrecht (Utrecht, The Netherlands).

### Reagents

PSM peptides were synthesized with the recently published sequences [7] by Genscript at 95% purity. PSMα1 (MGIIAGIIKVIKSLIEQFTGK), PSMα2 (MGIIAGIIKFIKGLIEKFTGK), PSMα3 (MEFVAKLFKFFKDLLGKFLGNN), PSMα4 (MAIVGTIIKIIKAIIDIFAK), PSMβ1 (MEGLFNAIKDTVTAAINNDGAKLGTSIVSIVENGVGLLGKLFGF), PSMβ2 (MTGLAEAIANTVQAAQQHDSVKLGTSIVDIANGVGLLGKLFGF), δ-toxin (MAQDIISTISDLVKWIIDTVNKFTKK) were all synthesized with an N-terminal formyl methionine residue. Peptide stocks were prepared at 2 mM dissolved in H2O except PSMα4, which was dissolved in 50% (v/v) MeOH/H20. Peptide grade TFA, and HPLC grade MeOH were purchased from Biosolve. fMLP, FITC-isomers, and C5a were obtained from Sigma Aldrich. IL-8 was purchased from PeproTech. HDL, LDL, VLDL and ApoB1 were purchased from (Millipore). Apo-A1 was purchased from (Calbiochem). PVL components LukS and LukF were kindly provided by Gerard Lina, Centre National de Référence des Staphylocoques, Lyon, France.

### Bacterial strains

In this study the *Staphylococcus aureus* strains COL, N315, MRSA252, Newman, MW2 and USA300 were used. MW2 *agr* knockout [35] was a kind gift of Alexander Horswill, the University Of Iowa, Iowa, USA. *S. aureus* strains were cultured overnight in Müller-Hinton broth (MHB) with shaking at 37°C. Alternatively, MW2 was grown in MHB until an OD_660_ of 1.0. Bacteria were washed with PBS and 10^6^ CFU/ml was added to freshly drawn whole human blood anticoagulated with 50 µg/ml lepirudin (Refludan, Schering). Bacteria were cultured overnight in whole blood with shaking at 37°C. Bacterial culture supernatants or whole blood culture plasma were clarified by centrifugation, filtered through 0.22 µm pore size filter and stored at −20°C in aliquots until use.

### Human neutrophils

Human neutrophils were isolated by means of the Ficoll-Histopaque gradient method. Venous heparinized blood was diluted with an equal volume of PBS, and subsequently layered on a gradient of Ficoll (Amersham Biosciences) and Histopaque (Sigma Aldrich). After centrifugation for 20 min at 400 g and 21°C, polymorphonuclear cells (neutrophils) were collected from Ficoll and Histopaque interfaces. Cells were washed with cold RPMI-1640 containing 25 mM HEPES, L-glutamine (Biowhittaker), and 0,05% human serum albumin (HSA; Sanquin) (RPMI-HSA). Erythrocytes were lysed by applying a hypotonic shock to the neutrophil pellet with distilled H_2_O for 30 sec, followed by 10× concentrated PBS to restore the isotonicity. The cells were washed and resuspended in RPMI-HSA. HL-60 cells stable transfected with the FPR2 (HL-60/FPR2), were kindly provided by F. Boulay (Laboratoire Biochimie et Biophysique des Systemes Integres, Grenoble, France). Cells were cultured in RPMI-1640 supplemented with 10% fetal bovine serum (FCS), 2 µm, 100 units/ml penicillin, 100 µg/ml streptomycin, and 600 µg/ml G418.

### Serum and lipoprotein preparations

Human pooled serum was obtained from at least 20 healthy donors and stored until use at −70°C. Serum was inactivated by heating at 56°C for 20 min. Isolation of lipid free serum was performed as described [1]. Briefly, EDTA-plasma or clarified lepuridin-plasma was applied on a gradient of potassium bromide and ultracentrifuged at 166.000 g for 22 h at 4°C. The lipid free serum fraction was isolated from the gradient with a density heavier than 1.25. The lipid fractions with a density between 1.063 and 1.210 and between 1.019 and 1.063 were used as the fractions containing HDL and LDL, respectively. Fractions were dialyzed against PBS, filtered (0.22 µm) and stored at 4°C until use. The concentration of HDL and LDL is expressed as the equivalent concentration of cholesterol in micrograms per milliliter. The purified lipoprotein and lipid free serum fractions obtained from the clarified lepirudin plasma were subject to a HPLC analysis. The lipid free serum fraction from the EDTA-plasma was subjected to a hexane extraction to remove the remaining lipid particles from the serum. The serum fraction and hexane 3∶1 (v/v) were incubated for 30 min while vigorously shaking. The aqueous partition was dialyzed against PBS. The protein content was measured with a standard BCA kit and concentrated with an Amicon 10 kDa cut-off filter (Millipore) to adjust the protein content to the level present in normal human serum.

### Calcium mobilization in human neutrophils and HL-60 cells

Calcium mobilization with isolated human neutrophils and HL-60/FPR2 cells was performed as previously described [13]. For this purpose, cells (5×10^6^ cells/ml) were loaded with 2 µM Fluo-3-AM for 20 min at room temperature, protected from light with gentle agitation. The cells were washed, resuspended in RPMI-HSA (without FCS) to 5×10^6^ cells/ml. Stimuli were prepared by incubating 25 µl of 10 times concentrated agonist with 25 µl 10 times concentrated heat inactivated serum, HDL, LDL or buffer for 30 min at room temperature. Before stimulation, cells were diluted to 1×10^6^ cells/ml in a volume of 200 µl. The basal fluorescence level for Fluo-3 was monitored at 530 nm for 8 sec after which 50 µl of pre-incubated stimulus was added. The sample tube was rapidly placed back to the sample holder and the fluorescence measurement continued up to 52 sec. Cells were gated based on scatter parameters to exclude cell debris and the mean fluorescence value at basal level was subtracted from the value at peak level (at 30 sec). The different fluorescent values were expressed as percentage of the maximal response for each individual stimulus. Alternatively, various concentrations of culture supernatants or synthetic PSM, pre-incubated with 1% or 0,1% heat inactivated serum for 30 min, were added to Fluo-3-labeled HL-60/FPR2 cells followed by flow cytometry. For inhibition kinetics, 10 times concentrated PSM were incubated with 0.1% serum and at different time-points of incubation samples were added as stimuli for HL-60/FPR2 cells in the flow cytometer.

### Neutrophil lysis assay

Lysis of human neutrophils by filter-sterilized *S. aureus* culture supernatants or synthetic PSM was measured as described [36], [37]. Clarified culture supernatants were pre-incubated with different concentrations of human serum for 10 min at room temperature. Pre-treated supernatants were transferred to a 96-wells ELISA plate (Nunc) containing 3×10^6^ neutrophils in a total volume of 100 µl RPMI-HSA and were incubated for 15 min at 37°C. Neutrophil lysis was determined by release of lactate dehydrogenase (LDH) using the CytoTox 96 Non-Radioactive Cytotoxicity kit (Promega). Alternatively, synthetic PSM were incubated for 10 min at room temperature with serum, serum gel filtration fractions or lipoproteins and subsequently tested in the neutrophil lysis assay.

### Gel filtration of PSM-FITC with serum

To study the PSM interaction with serum components, synthetic PSMα3 was labeled with fluorescein isothiocyanate (FITC), by incubating 1 mg/ml PSMα3 with 100 µg/ml FITC in 0.1 M sodium carbonate buffer (pH 9.6) for 1 hour at 37°C. FITC-labeled PSMα3 (PSMα3-FITC) was separated from unbound FITC using a HiTrap desalting column (Amersham Biosciences). To determine the retention volume of PSMα3 or PSMα3-FITC alone, 100 µg/ml was loaded onto a Superdex 200 10/300GL (GE Healthcare) equilibrated with PBS. The interaction with serum components was studied by incubating 100 µg/ml PSMα3-FITC with 10% human serum, 2 mg/ml HDL or 1 mg/ml LDL. In some experiments, 500 µl fractions were collected after column passage and fluorescence was quantified with a platereader fluorometer (Flexstation, Molecular Devices). Protein content was measured at OD280 nm and the FITC-extinction was measured at OD492 nm on an AKTA explorer (Amersham). Serum and lipoproteins had minimal auto extinction at OD492 nm allowing measurement of the association of PSMα3-FITC with serum components at OD492 nm. The column was calibrated using the HMW Calibration kit (GE Healthcare) containing Thyroglobulin (669 kDa), Ferritin (440 kDa), Aldolase (158 kDa), Conalbumin (75 kDa) and Ovalbumin (43 kDa). In some experiments, 100% heat inactivated serum was separated by gel filtration and subsequently 0.5 ml fractions were collected for further analysis. In other experiments, 100 µg/ml PSMα3-FITC in PBS containing 0.1% sodium-desoxycholate (PBS-DOC) was applied to the gel filtration column equilibrated with PBS-DOC.

### Serum pull-down assays and proteomics

PSMα1 and PSMα3 were immobilized on CNBr-Sepharose (Amersham) fast flow according to the manufacturer's suggestions. The coupling density was 2.5 mg/ml of resin for both PSM. Following quenching of excess reactive groups with 1 M ethanolamine (pH 8.0) for 2 h at room temperature, the affinity resins were washed and stored as 50% slurries in PBS. To test for binding to serum proteins, heat inactivated human serum was diluted 1∶20 in PBS and mixed individually with 20 µl of each affinity resin in a total volume of 0.5 ml. After a 30 min incubation at room temperature under vigorous agitation, the resins were pelleted by centrifugation at 3500 g for 2 min, and washed five times with 1 ml of PBS or PBS containing 0.1% Tween20 (PBST). After the last wash, each resin was resuspended in 20 µl of 2× Laemmli sample buffer, mixed briefly, and heated at 95°C for 5 min. Following sample preparation, the proteins contained in each sample were separated by 12.5% SDS-PAGE and visualized by instant blue staining. For protein identification, the bands of interest were excised from the gel and subjected to in-gel proteolysis by trypsin as described by [38]. The resulting tryptic fragments were extracted, separated by capillary liquid chromatography (LC), and characterized by tandem mass spectrometry (MS/MS). Proteins were identified by comparing the observed fragmentation ion patterns against a data base of human proteins using the MASCOT software package.

### HPLC analysis

Analytical HPLC was performed using an automatic HPLC system (Shimadzu) with an analytical reversed-phase column, an UV detector operating at 214 nm with a flow rate of 0.75 mL/min. A Phenomenex Gemini C18 (110 Å, 5 µm, 250×4.6 mm) column was used. TFA buffers (buffer A: H_2_O∶MeOH, 95∶5, v∶v; buffer B: MeOH∶H_2_O, 95∶5, v∶v, both containing 0.1% TFA). Elution was effected with either a linear gradient from 100% A to 100% B over 60 min or a linear gradient from 40%A to 100%B over 45 min. The molecular mass in the respective peak was determined using electrospray mass spectrometry (ESI-MS), which was performed on a Thermo Finnigan LCQ DECA XP MAX ion trap mass spectrometer and respective PSM were identified and matched in both retention time and mass synthetic PSM. δ-toxin and PSMα2 were not separated in both systems.

### Generation of PSMα promoter-GFP construct

The PSMα promoter-GFP construct was made similar to the method described previously [39]. Shortly, 270 bp upstream of the PMSα1 start codon (excluding the SD sequence) was amplified by PCR using Phusion polymerase (Finnzymes), using the following primers 5′-AGAATTCGCATGCCTAACGTGTTATTCGTTTTAAACTTAT-3′) and 5′-GGATCCTCTAGATTTGCTTATGAGTTAACTTCATTGTA-3′ (Life Technologies) and chromosomal DNA from strain Newman as template. Purified PCR products were digested with *XbaI* and *EcoRI* (New England Biolabs) and ligated in the likewise digested shuttle vector pSK236-GFP-uvr [39]. The ligation mixture was introduced in *E. coli* Top10F′ using the CaCl_2_ method [40]. Colonies were checked for GFP expression using an ImageQuant LAS4000 (GE Healthcare Life Sciences), and positive clones were checked by restriction analysis and sequencing of the insert. Correct plasmids were introduced into *S. aureus* strain RN4220 by electroporation as described by [41], re-isolated, and introduced by electroporation in *S. aureus* MW2 and the MW2 Agr knockout.

### GFP reporter assays

To measure the GFP expression of the *S. aureus* strains MW2 the MW2 agr KO in culture, the strains containing the PSMα promoter-GFP construct were grown in MHB with 10 µg/ml chloramphenicol. Overnight cultures were diluted 1∶10000 and grown to an OD_660_ of 0.1. The cultures were transferred to a clear 96 well flat bottom polystyrene tissue culture plates (Greiner) using 150 µl culture/well. The plate was grown in a Fluostar Omega plate reader (BMG labtech) at 37°C with constant double orbital shaking (400 rpm) in between measurements. Both the absorbance at 660 nm and GFP fluorescence (excitation 485 nm/emission 520 nm) were measured every 10 minutes for each well. The signal from 4 identical wells was averaged and corrected for blank wells containing only medium.

To detect GFP expression in neutrophils after phagocytosis of *S. aureus*, an overnight culture of *S. aureus* MW2, containing the PSMα promoter-GFP construct, was diluted 10000× in MHB and allowed to grow till OD_660_ 0.08. Then, the bacteria were collected by centrifugation and washed once in PBS. Bacteria were opsonized in 10% human serum in RPMI for 5′ at 37°C, washed once in PBS and resuspended in RPMI/HSA to an OD_660_ of 0.01, which corresponds to 5×10^6^ bacteria/ml. A three-channel flow cell for inverted microscopes (24×50 mm borosilicate cover glass, size 1,5 (VWR International BV, The Netherlands) mounted at the underside) with channel dimensions of 1×4×40 mm was assembled and sterilized as described [42]. To promote adherence of neutrophils, the channels were coated with 25% human serum in RPMI by flowing in the serum, stopping the flow, clamping off the channels on both sides, and incubating overnight at 4°C. Before introduction of neutrophils, the channels were flushed with RPMI to remove unbound serum. Freshly isolated neutrophils were diluted to 5×10^5^/ml and 200 µl was injected into each channel of the serum-coated flow cell. Neutrophils were allowed to adhere for 30 minutes at RT, after which the opsonized bacteria were injected in the channel at a ratio of 10 bacteria: 1 neutrophil. The channel was clamped on both sides, the tubes were cut and the flow cell was transferred to the microscope stage. Neutrophils and bacteria were imaged using a Leica TSC SP5 inverted microscope equipped with a HCX PL APO 40×/0.85 objective (Leica Microsystems, The Netherlands). The microscope was encased in a dark environment chamber that was stably kept at 37°C. 15 minutes after phagocytosis images were acquired using the camera every 5 minutes in both the bright field and the GFP channel (I3 filter cube) for 3 hours to follow GFP production. In post processing the bright field image was not altered and the green channel was adjusted in LAS AF (Leica) to contrast +10, brightness −10 and gamma 1.3 to reduce the green background before merging of both channels.

## Supporting Information

Figure S1 **Inhibition of PSM-related functions in culture supernatants by human serum.** (A) Dose-dependent calcium mobilization of HL-60/FPR2 cells by the culture supernatants of *S. aureus* strains USA300 Newman, MW2, COL and N315 with or without preincubation in 1% heat inactivated human serum. (B) Dose-dependent neutrophil lysis by *S. aureus* culture supernatants with or without 5% human serum. Neutrophil lysis was measured via LDH release. Data represent means ± SEM of three independent experiments.
(TIF)

Figure S2 **Inhibition of PSM-mediated PBMC lysis and no inhibition of PVL-mediated neutrophil lysis by human serum.** (A) Dose-dependent PBMC lysis by synthetic PSMα3 preincubated with or without 1% or 10% human serum. (B) Dose-dependent neutrophil lysis by recombinant PVL protein (LukS and/or LukF) with and without 5% serum. Leukocyte lysis was measured via LDH release. Data represent means ± SEM of three independent experiments.
(TIF)

Figure S3 **Calcium mobilization of human neutrophils induced by PSMα3 or PSMα3-FITC.** Neutrophils were stimulated with a dose response of 1.3×10^−9^ M to 10^−6^ M PSMα3 or PSMα3-FITC. Data represent means ± SEM of three independent experiments.
(TIF)

Figure S4 **Monomerization of PSM by serum lipoproteins.** Gel filtration association assay. Comparison of extinction (OD280 nm) profiles of 100 µg/ml PSMα3-FITC pre-incubated (A) 10% human serum, (B) 1 mg/ml HDL, (C) 1 mg/ml LDL or (D) 1 mg/ml VLDL for 30 min, before separation on a gel filtration column. Representative figures of two independent experiments.
(TIF)

Figure S5 **No inhibition of neutrophil activation or lysis by recombinant apolipoproteins.** (A) Dose-dependent neutrophil activation by synthetic PSMα3 preincubated with ApoA1 (50 µg/ml) or ApoB1 (50 µg/ml) or buffer, calcium mobilization was measured by flow cytometry. (B) Dose-dependent neutrophil lysis synthetic PSMα3 preincubated with ApoA1 50 µg/ml or ApoB1 50 µg/ml or buffer. Neutrophil lysis was measured via LDH release. Data represent means ± SEM of three independent experiments.
(TIF)

Video S1 **Time lapse images showing neutrophils which have phagocytozed** ***S. aureus*** **MW2 containing the PSMα promoter-GFP construct, PSMα expression (green) was monitored over time.** The video starts 15 min after phagocytosis and the images were taken by every 5 minutes. Note, the upper right corner shows a growing *S. aureus* reporter micro-colony which shows no PSM-α expression.
(MOV)
