# Supplementary figures and images for: Inactivation of Staphylococcal Phenol Soluble Modulins by Serum Lipoprotein Particles
Source: PLoS Pathog. 2012 Mar 22;8(3):e1002606. doi: 10.1371/journal.ppat.1002606 (PMC3310821; doi:10.1371/journal.ppat.1002606)

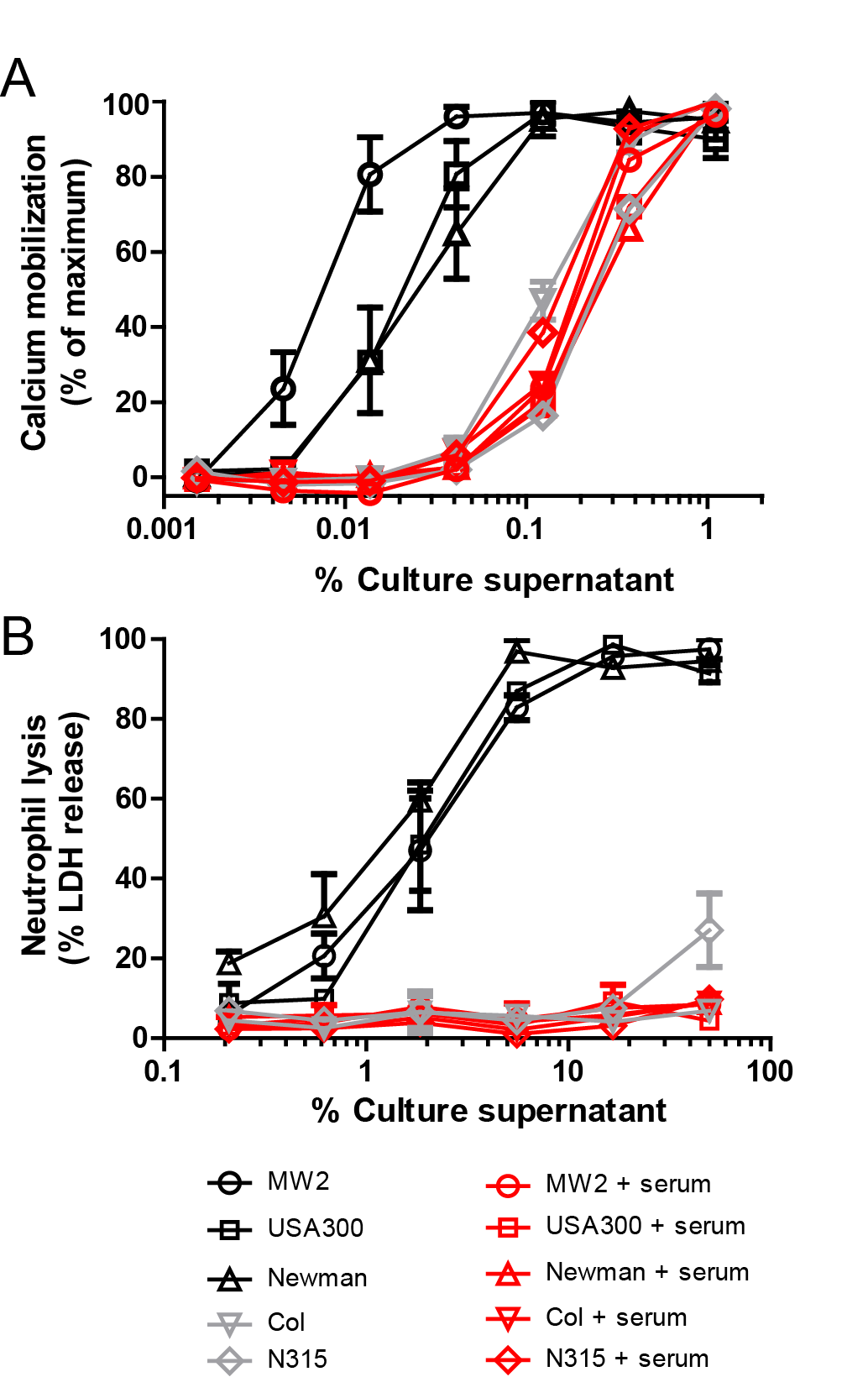

Supplement: Figure S1 — Inhibition of PSM-related functions in culture supernatants by human serum. (A) Dose-dependent calcium mobilization of HL-60/FPR2 cells by the culture supernatants of S. aureus strains USA300 Newman, MW2, COL and N315 with or without preincubation in 1% heat inactivated human serum. (B) Dose-dependent neutrophil lysis by S. aureus culture supernatants with or without 5% human serum. Neutrophil lysis was measured via LDH release. Data represent means ± SEM of three independent experiments. (TIF) [file ppat.1002606.s001.tif]

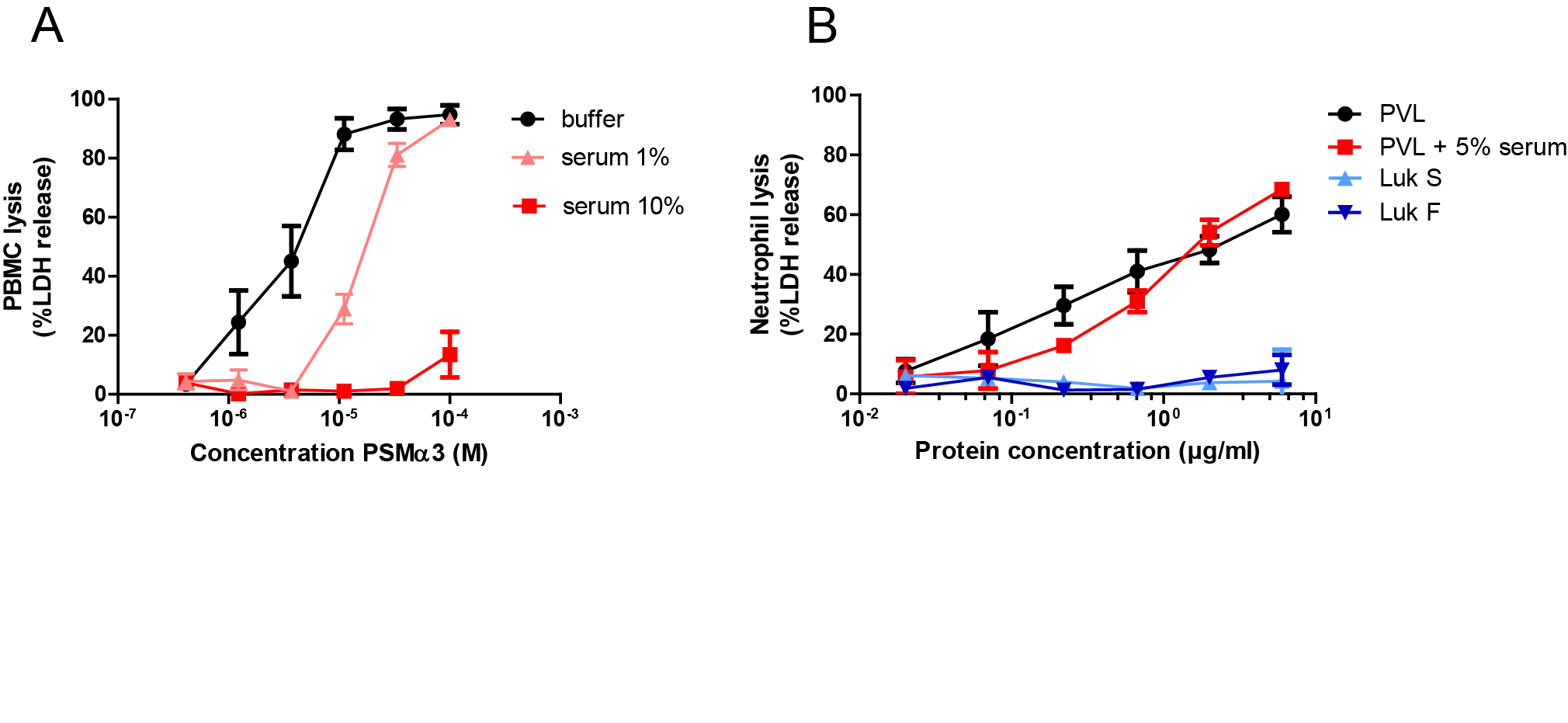

Supplement: Figure S2 — Inhibition of PSM-mediated PBMC lysis and no inhibition of PVL-mediated neutrophil lysis by human serum. (A) Dose-dependent PBMC lysis by synthetic PSMα3 preincubated with or without 1% or 10% human serum. (B) Dose-dependent neutrophil lysis by recombinant PVL protein (LukS and/or LukF) with and without 5% serum. Leukocyte lysis was measured via LDH release. Data represent means ± SEM of three independent experiments. (TIF) [file ppat.1002606.s002.tif]

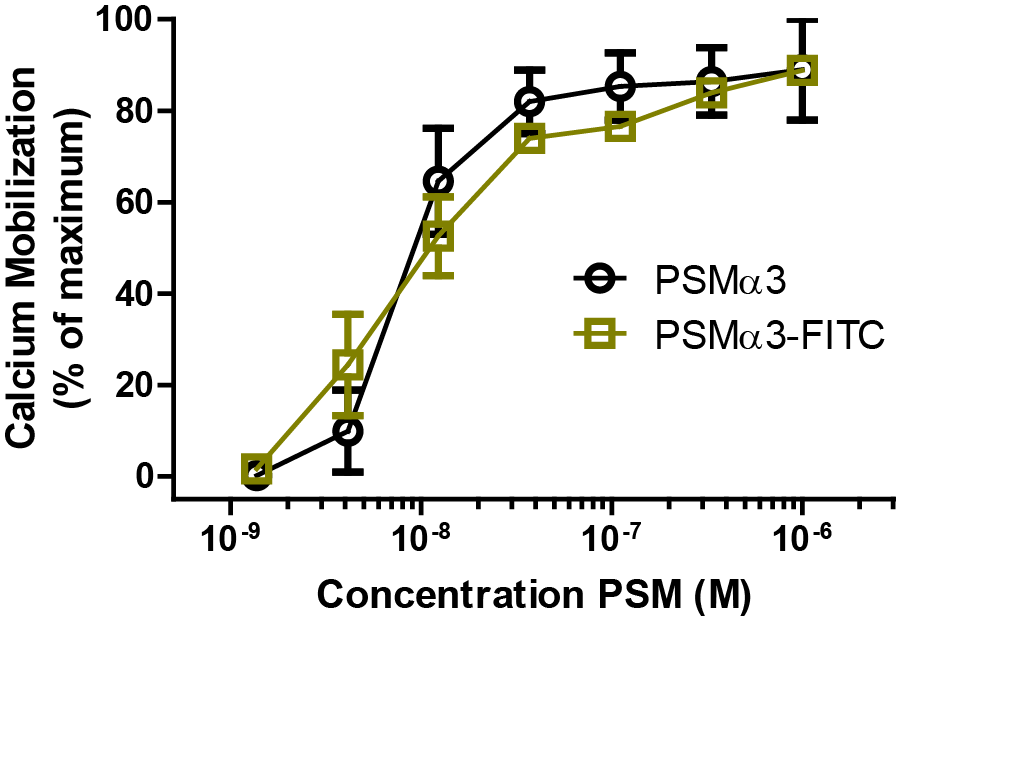

Supplement: Figure S3 — Calcium mobilization of human neutrophils induced by PSMα3 or PSMα3-FITC. Neutrophils were stimulated with a dose response of 1.3×10^−9^ M to 10−6 M PSMα3 or PSMα3-FITC. Data represent means ± SEM of three independent experiments. (TIF) [file ppat.1002606.s003.tif]

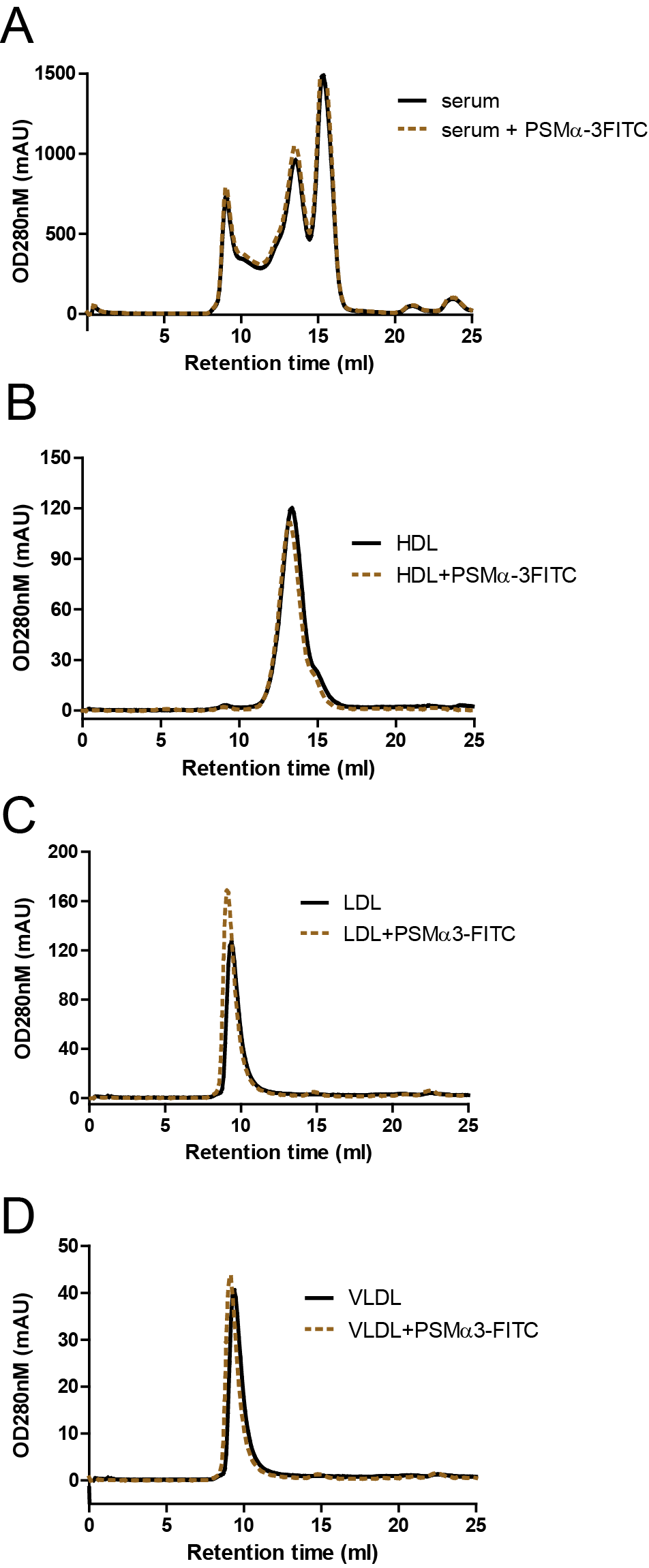

Supplement: Figure S4 — Monomerization of PSM by serum lipoproteins. Gel filtration association assay. Comparison of extinction (OD280 nm) profiles of 100 µg/ml PSMα3-FITC pre-incubated (A) 10% human serum, (B) 1 mg/ml HDL, (C) 1 mg/ml LDL or (D) 1 mg/ml VLDL for 30 min, before separation on a gel filtration column. Representative figures of two independent experiments. (TIF) [file ppat.1002606.s004.tif]

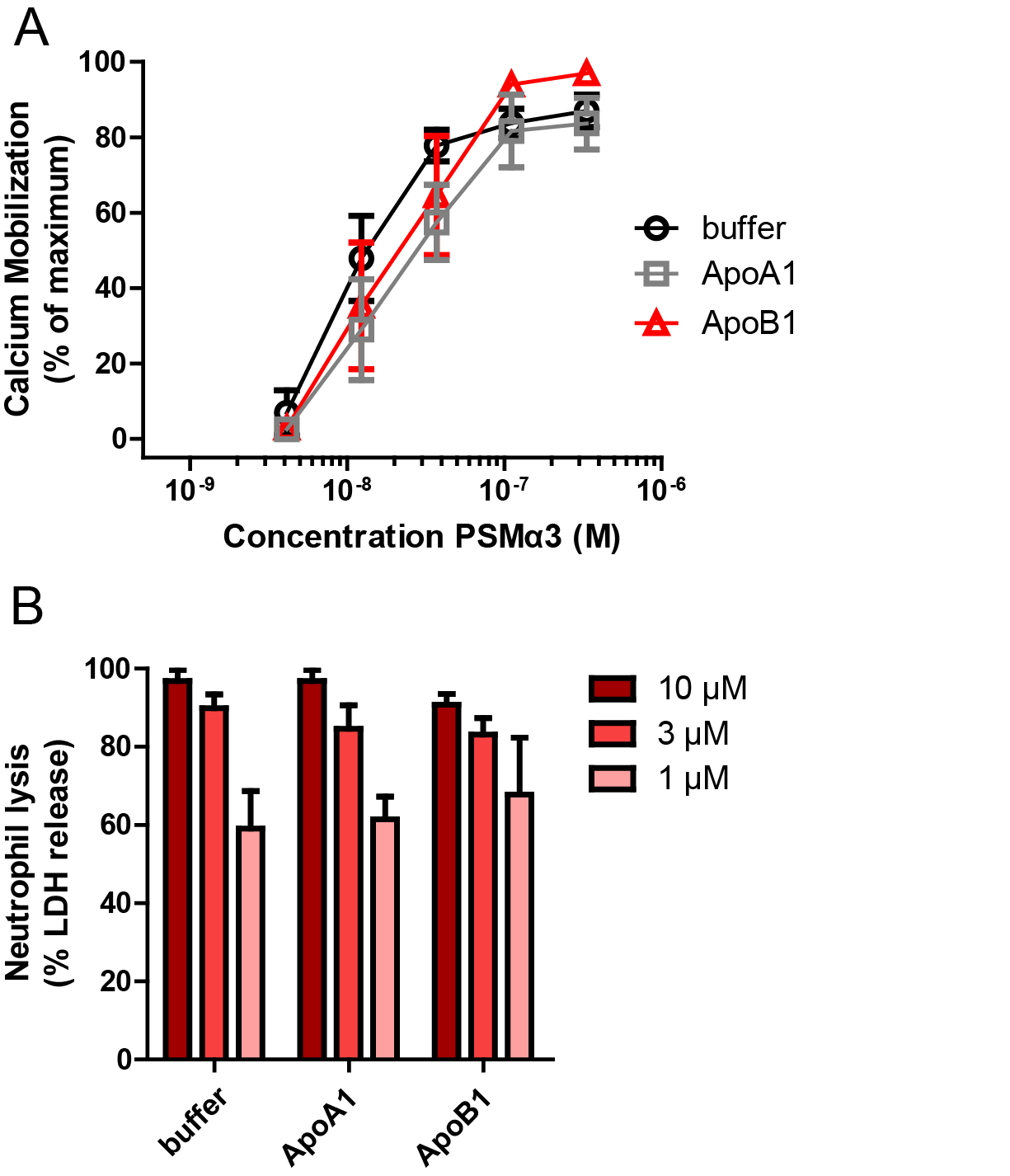

Supplement: Figure S5 — No inhibition of neutrophil activation or lysis by recombinant apolipoproteins. (A) Dose-dependent neutrophil activation by synthetic PSMα3 preincubated with ApoA1 (50 µg/ml) or ApoB1 (50 µg/ml) or buffer, calcium mobilization was measured by flow cytometry. (B) Dose-dependent neutrophil lysis synthetic PSMα3 preincubated with ApoA1 50 µg/ml or ApoB1 50 µg/ml or buffer. Neutrophil lysis was measured via LDH release. Data represent means ± SEM of three independent experiments. (TIF) [file ppat.1002606.s005.tif]
